# Glutathione mediated regulation of oligomeric structure and functional activity of *Plasmodium falciparum *glutathione S-transferase

**DOI:** 10.1186/1472-6807-7-67

**Published:** 2007-10-17

**Authors:** Timir Tripathi, Stefan Rahlfs, Katja Becker, Vinod Bhakuni

**Affiliations:** 1 Division of Molecular and Structural Biology, Central Drug Research Institute, Lucknow 226001, India; 2 Interdisciplinary Research Center, Justus-Liebig-University, Heinrich-Buff-Ring 26-32, 35392 Giessen, Germany

## Abstract

**Background:**

In contrast to many other organisms, the malarial parasite *Plasmodium falciparum *possesses only one typical glutathione *S*-transferase. This enzyme, *Pf*GST, cannot be assigned to any of the known GST classes and represents a most interesting target for antimalarial drug development. The *Pf*GST under native conditions forms non-covalently linked higher aggregates with major population (~98%) being tetramer. However, in the presence of 2 mM GSH, a dimer of *Pf*GST is observed. Recently reported study on binding and catalytic properties of *Pf*GST indicated a GSH dependent low-high affinity transition with simultaneous binding of two GSH molecules to *Pf*GST dimer suggesting that GSH binds to low affinity inactive enzyme dimer converting it to high affinity functionally active dimer. In order to understand the role of GSH in tetramer-dimer transition of *Pf*GST as well as in modulation of functional activity of the enzyme, detailed structural, functional and stability studies on recombinant *Pf*GST in the presence and absence of GSH were carried out.

**Results:**

Our data indicate that the dimer – and not the tetramer – is the active form of *Pf*GST, and that substrate saturation is directly paralleled by dissociation of the tetramer. Furthermore, this dissociation is a reversible process indicating that the tetramer-dimer equilibrium of *Pf*GST is defined by the surrounding GSH concentration. Equilibrium denaturation studies show that the *Pf*GST tetramer has significantly higher stability compared to the dimer. The enhanced stability of the tetramer is likely to be due to stronger ionic interactions existing in it.

**Conclusion:**

This is the first report for any GST where an alteration in oligomeric structure and not just small conformational change is observed upon GSH binding to the enzyme. Furthermore we also demonstrate a reversible mechanism of regulation of functional activity of *Plasmodium falciparum *glutathione *S*-transferase via GSH induced dissociation of functionally inactive tetramer into active dimers.

## Background

Development of resistance to the drugs used for prophylaxis and treatment of malaria makes the identification and characterization of novel drug targets necessary [1,2]. Glutathione S-transferases (GSTs^1^; EC 2.5.1.18) form a family of phase II detoxification enzymes and are present in virtually all organisms. GSTs catalyze the nucleophilic addition of the tripeptide glutathione to a large variety of nonpolar compounds, thereby neutralizing their electrophilic sites and rendering the products more water soluble [3]. These products can be more easily excreted, usually as a part of the mercapturic acid pathway [4]. GSTs can furthermore detoxify lipid peroxidation products [5,6] and serve as carrier proteins, so called ligandins, of certain organic molecules, which lead to the inactivation and immobilization of these compounds [4]. Other functions include the detoxification of hydroperoxides and the isomerization of specific metabolites (like prostaglandin H and 13- cis retinoic acid), reduction of dehydroascorbate and transfer of thiols [7] contributing to cellular signaling, regulation of transcription and stress response [8]. In cancer chemotherapy, the ability of GST to produce reactive metabolites has been exploited to target tumors that overexpress particular transferases [9].

On the basis of sequence similarity, immunological cross reactivity and substrate specificity, the cytosolic GSTs have been grouped into at least 13 classes: alpha, mu, pi, theta, sigma, kappa, omega, lambda, phi, tau, delta, zeta, and dehydroascorbate reductase (DHAR) [4,8,10-13]. Although the recognition of glutathione is conserved among different classes, the active site residues involved in glutathione binding vary from tyrosine in alpha, mu and pi classes (mammals) to serine in theta and zeta (ubiquitous), phi and tau (plants), and delta (insects) classes or cysteine in omega (mammals & insects), beta (bacteria) and lambda classes as well as in DHAR (plants) [13].

GST of the malarial parasite *Plasmodium falciparum *represents a novel GST isoform, which cannot be assigned to any of the known GST classes and constitutes more than 1% of the total cellular protein of the parasite [14,15]. *Pf*GST shares highest sequence similarities with pi-class GSTs from *Dirofilaria immitis *and *Onchocerca volvolus *(~35% identity). GST activity has been reported in all *Plasmodium *species studied so far as well as in all intraerythrocytic stages of the parasite [16]. Furthermore GST activity was shown to increase in chloroquine resistant parasites under drug pressure [17]. Ferriprotoporphyrin IX, which is produced in large quantities during the digestion of hemoglobin by the parasite, has been characterized as uncompetitive inhibitor of *Pf*GST [14]. A pharmacologic inhibition of *Pf*GST is likely to impair the peroxidase, conjugation and heme binding activity of the enzyme. This – together with the unique structural features of the enzyme – makes *Pf*GST, a most promising antimalarial drug target.

Conventionally the canonical GSTs are homo- or hetero- dimeric proteins with each subunit consisting of a thioredoxin like domain I fused to an all α-helical domain II [8]. The active site is located at the cleft between the two domains, consisting of two binding sites: the G-site which binds reduced glutathione and the more variable H-site, which can accommodate a variety of substrates [18]. The H-site of *Pf*GST differs significantly from that of its human counterpart in being more accessible to solvent molecules and amphiphilic inhibitors [18]. Aim of the present study was to investigate the role of GSH in tetramer-dimer transition of *Pf*GST, as well as in modulation of functional properties of the enzyme together with structural, activity and stability characterstics of the enzyme. Using size exclusion chromatography and protein cross-linking as well as fluorescence and CD spectroscopy in combination with enzyme activity assays we studied this unique feature of *Pf*GST in detail. The structural and functional characterization of *Pf*GST does contribute to our understanding of the parasites' biology; the evolution of GSTs and to targeted drug development against malaria.

## Results and discussion

### *Pf*GST is stabilized as a tetramer

**Figure 1:**
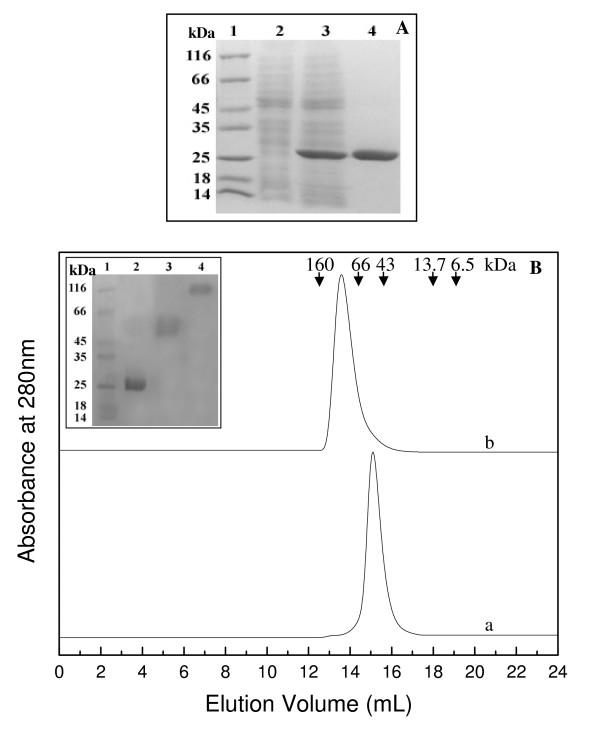
**Overexpression of *Pf*GST in *E. coli *and purification of the recombinant protein over Ni-NTA agarose**. A. SDS-PAGE analysis of cell lysate showing overexpression of *Pf*GST and the purified protein. Lanes 1–4 represent molecular weight markers, supernatant of un-induced culture lysate, supernatant of induced culture lysate and purified protein, respectively. B. Molecular weight andsubunit structure of *Pf*GST dimer and tetramer. SEC profileof (a) dimeric *Pf*GST; obtained by incubation of protein with 2 mM GSH and run with buffer containing 2 mM GSH and (b) tetrameric *Pf*GST; incubated and run with buffer not containing GSH on a Superdex™ 200 10/300 GL column at pH 8.0 and 25°C. The column was calibrated with standard molecular weight markers: Glucose oxidase (160 kDa), albumin (66 kDa), ovalbumin (43 kDa), ribonuclease A (13.7 kDa) and aprotinin (6.5 kDa). The curves have been displaced on Y-axis for presentation. Inset shows the SDS-PAGE profile of glutaraldehyde cross-linked *Pf*GST protein samples. Lanes 1–4 represent molecular weight markers, uncross-linked native *Pf*GST, glutaraldehyde cross-linked dimeric *Pf*GST and tetrameric *Pf*GST protein samples, respectively.

We carried out detailed studies on the structural and functional properties of *Pf*GST. Figure 1A shows the overexpression and purification of *Pf*GST, which had the expected molecular mass of about 26 kDa and a purity of about 95%. The quaternary structure of *Pf*GST was analyzed by SEC and glutaraldehyde cross-linking. On the Superdex™ 200 column the protein showed a single peak with a retention volume of 13.7 mL which corresponds to a molecular mass of 104 kDa, as calculated from the values obtained for the protein standards (Figure 1B). This suggests that the *Pf*GST is indeed present as a tetramer. This observation was further confirmed by the glutaraldehyde cross-linking of *Pf*GST, which showed the presence of a cross-linked protein band of a molecular mass of about 104 kDa corresponding to the tetramer of the enzyme (Figure 1B, inset). These results confirm previous experiments, which showed that recombinant *Pf*GST forms under native conditions non-covalently linked higher aggregates corresponding to 98% to tetramers [19]. To our knowledge *Pf*GST is the only enzyme of the GST family, which is present as a tetramer rather than a dimer.

### Glutathione binding to *Pf*GST modulates the conversion of inactive tetramer to active dimer

GSTs catalyze the general reaction GSH + R-X → GSR + H-X. GST brings the hydrophobic or amphiphilic substrate into close proximity with GSH and activates the sulfhydryl group of GSH thereby allowing the nucleophilic attack on the electrophilic substrate [20]. All GSTs are highly specific towards GSH as the thiol substrate. GSH is bound in an extended conformation to the enzyme at the G-site via a network of specific polar interactions between the tripeptide and a number of protein moieties in domain I of one subunit and one or two amino acid residues in domain II of the other subunit of the dimer. Hence the dimer of the enzyme is the active conformation. As all GSTs for which this catalytic mechanism has been described are dimers we wanted to study if GSH binds to the tetrameric protein and if this tetramer is functionally active.

**Figure 2:**
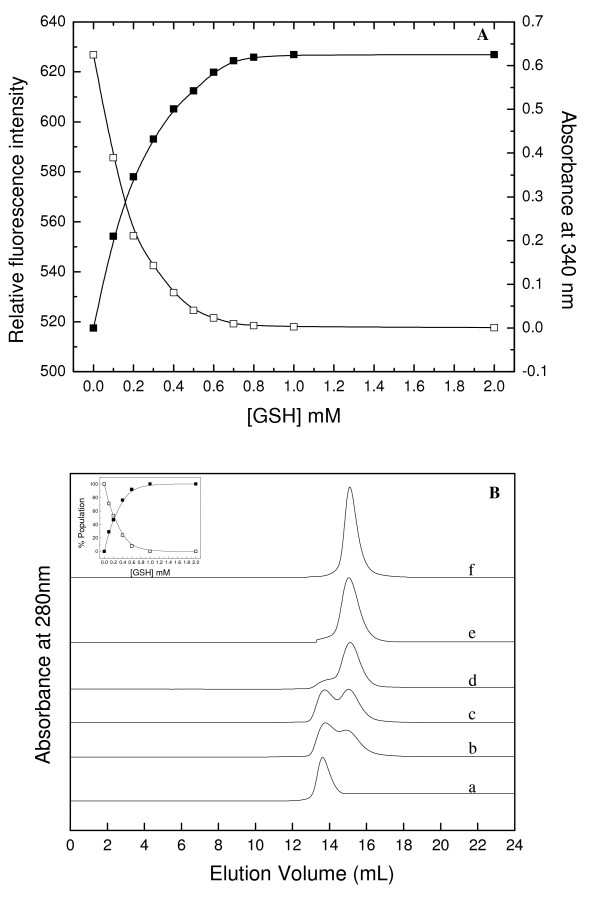
**Effect of GSH on oligomeric status and enzymatic activity of *Pf*GST**. A. Quenching of tryptophan fluorescenceintensity and enzymatic activity of tetrameric *Pf*GST withincreasing concentrations of GSH. In the figure, solid and hollow squares denote data for enzymatic activity and fluorescence, respectively. B. SEC profile of tetrameric *Pf*GST incubated with increasing concentrations of GSH for 2 h at 25°C and run in the same GSH containing buffer. In the figure, a-f represent curves for tetrameric *Pf*GST incubated with 0, 0.1, 0.2, 0.4, 0.6 and 2.0 mM GSH, respectively. The curves have been displaced on Y-axis for presentation. Inset shows the percent population of dimeric (solid squares) and tetrameric (hollow squares) species of *Pf*GST with increasing concentration of GSH based on the data obtained from the curves displayed in the main figure.

The binding of GSH to *Pf*GST can be investigated by monitoring the intrinsic tryptophan fluorescence of the enzyme. The substrate binding results in partial quenching of the fluorescence due to direct interactions between the bound substrate and the indole fluorophore of the tryptophan moiety [19,21]. We monitored the tryptophan fluorescence of the *Pf*GST tetramer incubated with increasing concentrations of GSH. Quenching of the tryptophan fluorescence intensity was observed at GSH concentrations between 0 to 0.8 mM (Figure 2A). Further increase in GSH concentration did not lead to any further quenching of fluorescence. However, a maximum of only about 20% quenching of the tryptophan signal was observed under these conditions. No alteration in the emission wavelength maxima of the tryptophan fluorescence was observed in the presence of GSH. These observations collectively suggest that in presence of GSH the *Pf*GST tetramer undergoes structural change.

A very interesting result was obtained when we studied the effect of GSH binding on the oligomeric structure of the enzyme. Figure 2B shows the SEC profiles of the *Pf*GST tetramer after incubation with increasing concentration of GSH. With increasing concentrations of GSH, two populations of the enzyme, one corresponding to the tetramer (retention volume 13.7 mL) and the other one corresponding to the dimer (retention volume 15.0 mL; molecular mass about 52 kDa) were observed. Furthermore, with increasing concentrations of GSH an enhancement in the area under the peak corresponding to the dimer along with a concomitant decrease in that corresponding to the tetramer were observed. At GSH concentrations ≥ 0.7 mM only a single peak corresponding to the dimeric protein was observed. This was further confirmed by the appearance of a cross-linked protein band of about 52 kDa (Figure 1B, inset). Furthermore, this GSH dependent tetramer to dimer transition was found to be independent of the enzyme concentration (data not shown). These observations demonstrate that GSH binding to the *Pf*GST tetramer induces a dissociation of the tetramer into dimers and that in the presence of higher GSH concentrations a GSH-stabilized dimer is obtained.

As GSH was shown to induce dissociation of the native *Pf*GST tetramer into dimers we carried out activity assays in presence of increasing concentrations of GSH to see which configuration, dimer or tetramer, was functionally active. Figure 2A shows the functional activity of the *Pf*GST tetramer in the presence of increasing concentrations of GSH. An exponential increase in enzymatic activity of *Pf*GST was observed at GSH concentrations between 0 and 0.7 mM. However, further increase in GSH concentration did not lead to further enhancement in activity. The results demonstrate that the conditions for attainment of maximal activity, namely saturation of GSH binding to the *Pf*GST tetramer as well as complete dissociation into dimers all occur at the same GSH concentration. This demonstrates that the dimer and not the tetramer of *Pf*GST is the functionally active form of the enzyme. The fact that the increase in activity is directly paralleled by the dissociation of the tetramer indicates that the K_m _values determined do not simply reflect the affinity of GSH to the active enzyme but at the same time induce major changes in quaternary structure resulting in active enzyme. This is the first report for any GST where an alteration in oligomeric structure and not just small conformational change are observed upon GSH binding.

**Figure 3:**
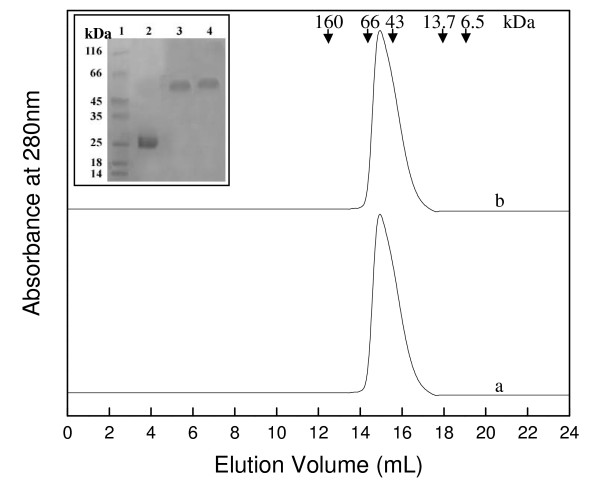
**Effect of GSH on oligomeric status of human GST**. SEC profile of human GST. Curve "a" represent *h*GST incubated and run with buffer containing 2 mM GSH, while curve "b" represent *h*GST incubated and run with buffer not containing GSH. The curves have been displaced on Y-axis for presentation. Inset shows the SDS-PAGE profile of glutaraldehyde cross-linked *h*GST protein samples. Lanes 1–4 represent molecular weight markers, uncross-linked native *h*GST, glutaraldehyde cross-linked *h*GST incubated with GSH and *h*GST incubated without GSH protein samples, respectively.

In direct comparison with *Pf*GST we studied the effect of GSH on the oligomeric status of human GST. Figure 3 shows the SEC profiles of human GST in presence and absence of GSH. For human GST a single peak with a retention volume of 15.1 mL corresponding to a molecular mass of about 52 kDa was observed which clearly represents the dimeric protein. For the enzyme sample incubated with GSH and run in presence of GSH no shift in retention volume from the column was observed. This was further confirmed by cross-linking of the eluted peaks (Figure 3 inset). This demonstrates that human GST is present as a dimer under physiological conditions and binding of GSH to the human GST dimer does not affect the oligomeric status of the enzyme.

From the recently reported crystal structure of *Pf*GST, it has been deduced that in absence of ligands two biological dimers form a tetramer [18]. In the tetramer the homodimers are interlocked with each other by a loop interacting with the substrate-binding site (residues 113–120). Upon binding of S-hexylglutathione, this loop undergoes rearrangement. The changed course of residues 113–120 in the liganded enzyme prevents locking of the dimers (19). A similar process is likely to take place upon GSH binding to the enzyme. The oligomerization of dimer into tetramer can be only a consequence and not the cause of the loss of affinity for GSH as the tetramer formation can be triggered by the structural change in the G-site of the enzyme in the absence of GSH.

### The GSH-induced dissociation of the *Pf*GST tetramer is a reversible process

**Figure 4:**
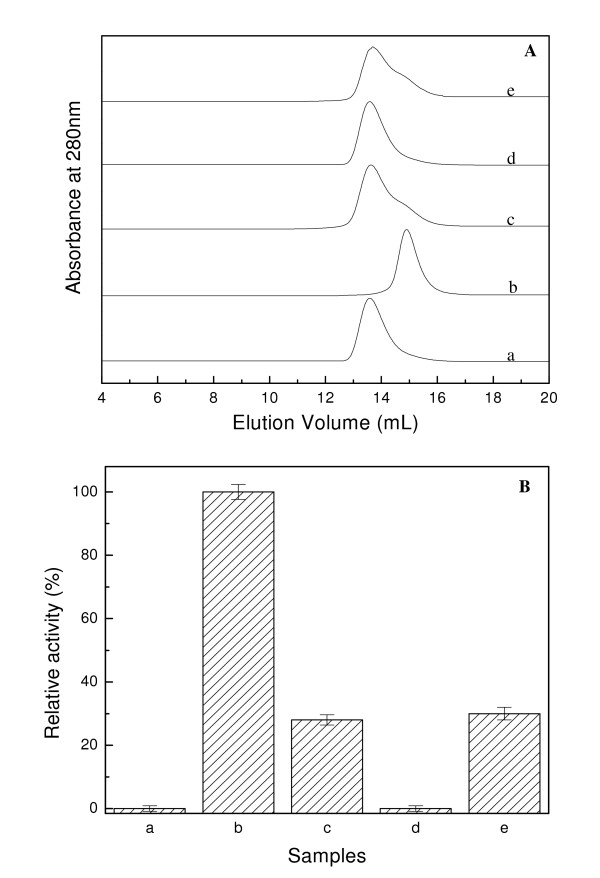
**Deciphering the reversibility of the tetramer-dimer transition**. A. SEC profile of *Pf*GST. Curves a-e represent tetrameric *Pf*GST, dimeric *Pf*GST, dimeric *Pf*GST dialysed for 4 h in buffer devoid of GSH, dimeric *Pf*GST dialysed for 24 h in buffer devoid of GSH, dimeric *Pf*GST loaded on the column equilibrated and run with buffer without GSH. The curves have been displaced on Y-axis for presentation. B. Enzymatic activity assay of the eluted peaks. In the figure, bars a-e represents data for the peaks described in 4A.

It has been reported that *Pf*GST shows good stability and constant specific activity only when it is stored at ≥ 1 mM GSH [21]. The specific activity of the enzyme stored in 10 mM GSH drops to 50% in a few minutes after SEC in absence of GSH or when the enzyme is stored in buffer where GSH is absent [21]. However, no reason for this partial or complete inactivation of *Pf*GST has been given so far. Our results indicate that GSH is essential for the conversion of enzymatically inactive tetramer into active dimer. It might thus be possible that the tetramer-dimer conversion of *Pf*GST is a reversible process, which is modulated by GSH. To study this hypothesis two sets of experiments were carried out. Firstly the tetrameric *Pf*GST incubated with 2 mM GSH was loaded on a Superdex™ 200 column and was run with buffer, which did not contain GSH. Figure 4A summarizes the results of this study. A SEC profile containing two populations, dimer and tetramer, of enzyme was observed under these conditions (Figure 4A(e)). This suggests that a significant population (about 75%) of the enzymatically active dimer is converted into inactive tetramer under these conditions and this is what was reflected in the activity profile where only about 30% activity was observed for the sample obtained after SEC (Figure 4B). In the second experiment, the GSH-stabilized enzymatically active dimer was dialyzed against the buffer in which GSH was absent. The dialyzed protein sample was loaded on the Sephadex S-200 column after 4 and 24 h of dialysis. Figure 4A(c, d) summarizes the results. For the 4 h dialyzed sample, the presence of both the dimer and the tetramer in a ratio of about 1:3 was observed. However, after 24 h of dialysis only the tetrameric enzyme species was observed. The analysis of the enzymatic activity of these two samples showed about 28% and 0% activity, respectively (Figure 4B). These studies clearly demonstrate that the GSH-induced tetramer to dimer conversion of *Pf*GST is a reversible process with the equilibrium shifting to either side due to the presence or the absence of GSH.

### Stability of *Pf*GST tetramer and dimer against pH-, GdnHCl- and urea-induced denaturation

**Figure 5:**
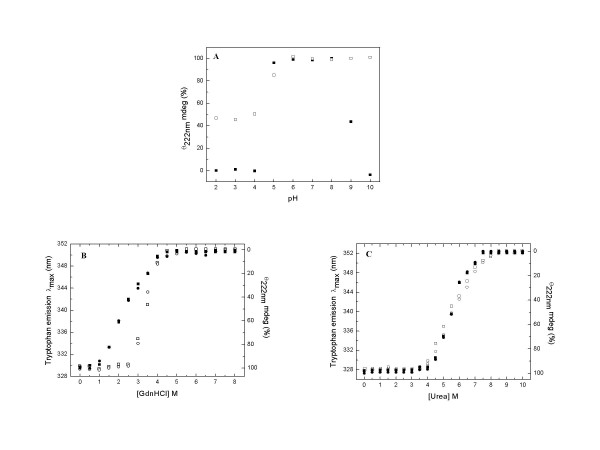
**pH-, GdnHCl- and urea- induced unfolding of dimeric and tetrameric *Pf*GST at pH 8.0 and 25°C**. A. Effects of the pH on the CD signal at 222 nm for the dimeric (solidsquares) and tetrameric (hollow squares) *Pf*GST. The data is presented as percentage with the value observed for enzyme at pH 8.0 taken as 100%. B Effect of increasing GdnHCl concentrations on the CD ellipticity at 222 nm and the tryptophan emission wavelength maxima of dimeric and tetrameric *Pf*GST. C. Effect of increasing urea concentrations on the CD ellipticity at 222 nm and the tryptophan emission wavelength maxima of dimeric and tetrameric *Pf*GST. In the panel B and C, solid squares, solid circles, hollow squares and hollow circles represent data for CD of the dimers, fluorescence of the dimers, CD of the tetramers and fluorescence of the tetramers, respectively. The CD data has been presented as percentage with the value observed in the absence of denaturant (GdnHCl or urea) taken as 100%. The experimental details are mentioned in the Methods section.

The effect of changing pH values on the structural features of the dimeric and tetrameric species of *Pf*GST was studied. Figure 5A shows the changes in secondary structure of dimeric and tetrameric *Pf*GST with varying pH. For the *Pf*GST dimer, the secondary structure was found to be stable between pH 5.0 and 8.0 as no alteration in secondary structure of the protein was observed. However, decrease in pH below 5.0 or increase in pH above 8.0 resulted in denaturation of the enzyme as indicated by a significant loss in secondary structure. At pH 4.0 or below and pH 10.0, almost complete loss of secondary structure was observed. For the *Pf*GST tetramer, a sigmoidal dependence of the loss of secondary structure on the pH value was observed. Between pH 10.0 and 6.0 no alteration was determined. However, decrease in pH below 6.0 resulted in loss of secondary structure. Interestingly, for the *Pf*GST tetramer even at a pH as low as 3.0, only a partial loss of secondary structure of about 50% was observed suggesting only partial unfolding of the enzyme at low pH.

The pH dependent studies demonstrate that unlike the dimer which is denatured at alkaline pH, the *Pf*GST tetramer is resistant to alkaline pH. Both the tetramer and the dimer show susceptibility to acidic pH, the tetramer is however more stable under these conditions. Taken together these observations demonstrate that the *Pf*GST tetramer is more stable than the dimer towards pH induced denaturation, which is likely to be due to stronger ionic interactions present in the tetrameric conformation.

The comparative unfolding and stability characteristics of *Pf*GST tetramer and dimer were studied by monitoring the GdnHCl-induced changes in the structural properties of the proteins. To study the changes in the secondary and tertiary structure induced by GdnHCl, far-UV CD and tryptophan fluorescence studies were carried out. A minimum time of about 2 h was found to be sufficient for achieving equilibrium under any of the denaturing conditions studied. Figure 5B summarizes the effect of increasing concentrations of GdnHCl on the CD ellipticity at 222 nm and tryptophan emission maxima of the *Pf*GST tetramer and dimer. For both conformations of the enzyme, a sigmoidal loss of the CD signal at 222 nm and a shift in the emission wavelength maxima of the tryptophan fluorescence from 328 nm to 353 nm was observed. Furthermore, the changes in tryptophan fluorescence and the secondary structure of the proteins were found to be concomitant with respect to GdnHCl concentration. However, the GdnHCl concentration over which the transition was observed was different for the two forms of the enzyme. For the *Pf*GST dimer, the transition occurred between 1.0 and 4.0 M GdnHCl, with C_1/2 _for the transition being 2.5 M GdnHCl. For the *Pf*GST tetramer the transition was observed between 2.5 and 4.0 M GdnHCl, with C_1/2 _for the transition being 3.5 M GdnHCl.

The comparative unfolding and stability characteristics of the *Pf*GST tetramer and dimer with urea were studied by monitoring the urea-induced changes in the structural properties of the proteins. Figure 5C summarizes the effect of increasing concentrations of urea on the CD ellipticity at 222 nm and the tryptophan emission maxima of *Pf*GST tetramers and dimers. For both conformations, a similar sigmoidal loss of the CD signal at 222 nm and a shift in the emission wavelength maxima of the tryptophan fluorescence from 328 nm to 353 nm was observed for CD and fluorescence spectroscopy, respectively. According to this data, a C_1/2 _of about 5.5 M urea was found to be associated with the unfolding of both the *Pf*GST tetramer and dimer.

These results demonstrate that the *Pf*GST tetramer has significantly higher stability against GdnHCl denaturation as compared to the *Pf*GST dimer. However, with respect to urea denaturation the tetramer and the dimer showed similar stability. These observations along with the pH stability studies suggest that the higher stability of the tetramer of *Pf*GST as compared to the dimer is probably due to the presence of stronger ionic interactions in this conformation of enzyme.

## Conclusion

The glutathione *S*-transferase of the malarial parasite *Plasmodium falciparum *is the first GST for which a GSH induced tetramer-dimer transition has been described. Interestingly, the surrounding GSH levels in a physiological concentration range of 0–0.7 mM regulate this transition. Until now the determination of GSH levels in malarial parasites has been restricted to late trophozoites, which represent the largest blood stage form and are thus accessible to studies. Meierjohann *et al*., described a variation in GSH levels between different strains [22]. For *Pf*Dd2 they measured 134 nmol/10^10 ^cells, for *Pf*3D7 67 nmol/10^10 ^cells. Assuming that a parasitized erythrocyte has 90 fL (90 × 10^-15 ^L) and 30 fL (corresponding to 1/3 of the host cell compartment as described in Atamna and Ginsburg [23]) of the cell is represented by the trophozoite parasite, 10^10 ^parasite cells would have a volume of 30 × 10^-5 ^L which is equal to 300 μL. 134 nmol/300 μL corresponds to 447 μM for *Pf*Dd2. For *Pf*3D7 a value of 223 μM can be calculated. In FCBR-strain, Luersen *et al*., found a GSH concentration of 0.42 mM [24]. Atamna and Ginsburg measured a GSH concentration of 790 nmol/10^10 ^cells in the strain FCR3, corresponding to 2.6 mM. This is by a factor of 10 higher than measured in the 3D7 strain. All these data indicate that the GSH concentrations may vary considerably between different strains. Furthermore, the reliable determination of intraparasitic GSH concentrations remains challenging – particularly due to the limited material and the complex isolation and washing procedures required. These are also the reasons why GSH levels throughout the complex life cycle of *Plasmodium *in man and mosquitoes have not yet been studied although they might vary considerably [25,26]. Taking into account GSH concentrations between 223 μM and 2.6 mM (see above) as well as the fact that GSH has not yet been determined in most of the parasite stages, a physiological role of *Pf*GST activity regulation by GSH in the micromolar range should be seriously considered.

**Figure 6:**
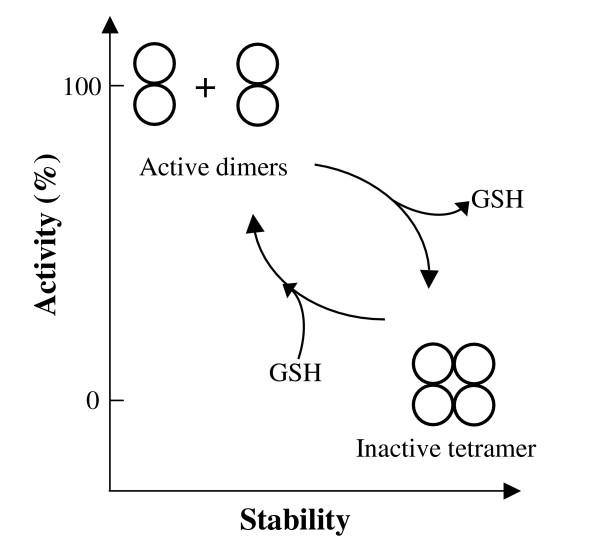
Schematic overview of *Pf*GST tetramer-dimer transition with respect to GSH binding, activity and stability.

It is thus possible that *Pf*GST is produced and – depending on the surrounding GSH concentration – present as inactive but more stable tetramer. With increasing GSH concentrations the enzyme becomes active, with decreasing GSH concentrations it tetramerises again (Figure 6) and this reversible transition regulates the functional activity of *Pf*GST. Under conditions of oxidative stress in the parasite, GSH levels can be depleted [25] and this would prevent the dissociation of *Pf*GST leading to accumulation of inactive tetramer. Other regulatory principles including low molecular weight ligands, post-translational modifications or protein-protein interactions might further contribute to the modulation of this phenomenon. Since *Pf*GST currently represents one of the most attractive antimalarial drug targets and the reversible changes in the oligomerization state of the protein is likely to reflect a novel principle of GST regulation, we will address this phenomenon in further studies.

## Methods

### Materials

All chemicals used in the study were purchased from Sigma-Aldrich Chemical Co., USA, and were of highest purity available. The *h*GST from human placenta was also obtained from Sigma. The water used for all studies was of triple distilled grade. Superdex™ 200 10/300 GL column was purchased from GE Healthcare Biosciences, USA, while Ni-NTA agarose was from Qiagen.

### Protein expression and purification

Recombinant *Pf*GST was overexpressed in *E. coli *M15 cells and purified as described earlier [14]. The ESI-MS and SDS-PAGE of the purified *Pf*GST protein showed that the preparation was more than 95% pure. The protein was dialyzed against 100 mM potassium phosphate buffer, pH 8.0 containing 1 mM EDTA. Two different buffer systems were used- containing or not containing 2 mM GSH.

### Size exclusion chromatography

Gel filtration experiments were carried out on a Superdex™ 200 10/300 GL column (manufacturer's exclusion limit 600 kDa for proteins) on an ÄKTA-FPLC (GE Health Care Biosciences). The column was equilibrated and run with 100 mM potassium phosphate buffer, pH 8.0 containing 1 mM EDTA, with a flow rate of 0.3 mL/min at 25°C. For GSH treated protein samples, the column was equilibrated and run with the above-mentioned buffer containing desired concentrations of GSH.

### Chemical cross-linking

The protein samples, at a concentration of 50 μg/mL, were used for cross-linking studies. An aliquot of 25% (w/v) glutaraldehyde was added to obtain a final glutaraldehyde concentration of 1%. The samples (2 mL) were stirred for 30 min at room temperature followed by quenching of the cross-linking reaction by the addition of 2 μL of β-mercaptoethanol. After incubation for 20 min, 3 μL of 10% aqueous sodium deoxycholate was added. The pH of the reaction mixtures was lowered to 2–2.5 by the addition of orthophosphoric acid, which resulted in precipitation of the cross-linked proteins. After centrifugation, the obtained precipitates were re-dissolved and the molecular mass of the cross-linked products was determined by 10% SDS-PAGE.

### Enzymatic activity

*Pf*GST activity using GSH and CDNB as substrates was determined spectrophotometrically at 340 nm on the basis of the extinction coefficient for the product *S*-(2, 4-dinitrophenyl) glutathione (ε_340 nm _= 9.6 mM ^-1^cm^-1^). The assay mixture (1 mL) contained 2.0 μM *Pf*GST enzyme and 1 mM GSH in 100 mM potassium phosphate buffer, pH 8.0, 1 mM EDTA. The reaction was started by addition of 0.5 mM CDNB. One unit of GST activity was defined as the conjugation of 1 μmol of CDNB with GSH per minute at 25°C [14]. The data was recorded with a Perkin-Elmer Lambda 25 UV/Vis spectrophotometer at 25°C.

### Fluorescence spectroscopy

Fluorescence spectra were recorded with a Perkin-Elmer LS50B spectrophotometer in a 5 mm path length quartz cell. 2.0 μM protein was used for the studies. The samples were excited at 285 nm and the emission spectra were recorded in the wavelength range of 300–400 nm. Spectral scans were repeated thrice and the average was taken. The excitation and emission slits were kept as 8 and 6 nm respectively. Data was recorded at 25°C.

### Far- ultraviolet circular dichroism

Far- UV CD measurements were made with a Jasco J-810 spectropolarimeter calibrated with ammonium(+)-10-camphor sulphonate. CD spectra were measured at an enzyme concentration of 2 μM with a 1 mm cell at 25°C. In a typical experiment 3 spectral scans were taken. The values obtained were normalized by subtracting the baseline recorded for the buffer having the same concentration of denaturant and/or GSH under identical conditions.

### pH denaturation

Protein (2 μM) was dissolved in 10 mM citrate/glycine/hepes buffer (containing 1 mM EDTA) of varying pH (from 2.0 to 10.0) in the absence or presence of GSH and was incubated for 2 h at room temperature (pH of the solution maintained) before the measurements were made.

### GdnHCl and urea denaturation

Protein (2 μM) was dissolved in 100 mM potassium phosphate buffer, pH 8.0 containing 1 mM EDTA in the absence or presence of increasing concentration of GdnHCl/urea with or without GSH. It was incubated for 2 h at room temperature, before the measurements were made.

## Abbreviations

*Pf*GST, *Plasmodium falciparum *glutathione S-transferase; *h*GST, human GST; GSH, Reduced glutathione; C_1/2_, Denaturant concentration at transition midpoint; CDNB, 1-chloro-2,4-dinitrobenzene; ESI-MS, Electrospray ionization mass spectrometry; SEC, Size exclusion chromatography; Ni-NTA, Nickel-nitrilotriacetic acid; GdnHCl, Guanidine hydrochloride.

## Competing interests

The author(s) declares that there are no competing interests.

## Authors' contributions

TT carried out all the experiments and the data analysis. SR, KB and VB conceived the study, participated in its design and coordination, and drafted the manuscript. All authors read and approved the final manuscript.
